# Loss of PDK1 Induces Meiotic Defects in Oocytes From Diabetic Mice

**DOI:** 10.3389/fcell.2021.793389

**Published:** 2021-12-20

**Authors:** Juan Ge, Na Zhang, Shoubin Tang, Feifei Hu, Xiaojing Hou, Hongzheng Sun, Longsen Han, Qiang Wang

**Affiliations:** ^1^ State Key Laboratory of Reproductive Medicine, Suzhou Municipal Hospital, Nanjing Medical University, Nanjing, China; ^2^ Department of Obstetrics and Gynecology, The Second Affiliated Hospital of Nanjing Medical University, Nanjing, China; ^3^ Women’s Hospital of Nanjing Medical University, Nanjing Maternity and Child HealthCare Hospital, Nanjing, China; ^4^ Center for Global Health, School of Public Health, Nanjing Medical University, Nanjing, China

**Keywords:** oocyte, meiosis, diabetes, spindle, PDK1

## Abstract

Maternal diabetes has been shown to impair oocyte quality; however, the underlying mechanisms remain unclear. Here, using a streptozotocin (STZ)-induced diabetic mouse model, we first detected and reduced expression of pyruvate dehydrogenase kinase 1 (PDK1) in diabetic oocytes, accompanying with the lowered phosphorylation of serine residue 232 on α subunit of the pyruvate dehydrogenase (PDH) complex (Ser232-PDHE1α). Importantly, forced expression of PDK1 not only elevated the phosphorylation level of Ser232-PDHE1α, but also partly prevented the spindle disorganization and chromosome misalignment in oocytes from diabetic mice, with no beneficial effects on metabolic dysfunction. Moreover, a phospho-mimetic S232D-PDHE1α mutant is also capable of ameliorating the maternal diabetes-associated meiotic defects. In sum, our data indicate that PDK1-controlled Ser232-PDHE1α phosphorylation pathway mediates the effects of diabetic environment on oocyte competence.

## Introduction

Women with diabetes often suffer from many reproductive problems such as infertility, miscarriage, and congenital malformations (Greene 1999; Wang and Moley 2010). Meantime, preovulatory oocytes from diabetic mice experience an increased incidence of spindle disorganization and chromosome congression failure, leading to the generation of aneuploid eggs (Chang et al., 2005; Ding et al., 2012; Wong et al., 2015; Liu et al., 2017). So far, the developmental abnormalities at multiple stages induced by maternal diabetes have been reported in animal and human studies (Amaral et al., 2008; Jungheim and Moley 2008). Mammalian oocytes undergo a long and discontinuous developmental process that begins in the fetal stage until adulthood, which has profound effects on fertilization, embryonic development and even adult disease (Sirard et al., 2006; Jungheim and Moley 2008). Hence, clarification of how diabetes influences oocyte development may uncover the origin of reproductive defects in diabetic mother.

Developmental competence of an oocyte requires energy production and active synthesis. Pyruvate utilization and oxygen consumption have been found to be gradually elevated throughout the period of oocyte growth in mice (Biggers et al., 1967; Zuelke and Brackett 1992; Saito et al., 1994); instead, glucose first needs to be converted to pyruvate by granulosa cells to support oocyte maturation (Eppig 1976; Fagbohun and Downs 1992). The pyruvate dehydrogenase (PDH) is a mitochondrial matrix multienzyme complex that provides the link between glycolysis and tricarboxylic acid (TCA) cycle by catalyzing the conversion of pyruvate into acetyl coenzyme A (acetyl-CoA) (Hou et al., 2015). In mammals, pyruvate dehydrogenase kinase (PDK) and pyruvate dehydrogenase phosphatase (PDP) co-regulate the PDH complex activity, through a reversible phosphorylation-dephosphorylation cycle. (Sugden and Holness 2003; McFate et al., 2008). Earlier studies revealed that PDH activity is suppressed by PDK in response to site-specific phosphorylation at three sites on the E1 alpha subunit of PDH (PDHE1a; Ser232, Ser293, and Ser300) (Korotchkina and Patel 2001; Rardin et al., 2009; Hitosugi et al., 2011). To date, genetically and biochemically distinct PDK family isozymes (PDK1, 2, 3, and 4) have been identified in mammalian species (Tokmakov et al., 2009). They have diverse tissue-specific distributions and specificities. PDK1 is expressed in heart and has been reported to be closely correlated with tumorigenesis (Patel and Korotchkina 2001; Roche and Hiromasa 2007; Ratchford et al., 2008). PDK2 can be detected in many tissues, whereas PDK3 is mainly found in testis. PDK4 is substantially expressed in heart and skeletal muscle (Gudi et al., 1995; Bowker-Kinley et al., 1998; Korotchkina and Patel 2001; Kwon et al., 2006). PDK1 is a critical glucose metabolism enzyme regulating glycolysis or glucose oxidase in cells (Yang et al., 2016). Our previous data demonstrated that PDK1/2 knockdown disturbs the assembly of meiotic apparatus during mouse oocyte maturation (Hou et al., 2015).

It has been reported that oocytes derived from diabetic mice display the meiotic abnormalities and metabolic dysfunction (Cheng et al., 2011). However, to date, the potential mechanisms remain to be explored. In the present study, by employing a streptozotocin (STZ)-induced diabetic mouse model, we investigated whether PDKs are involved in the compromised oocyte quality due to the exposure of maternal diabetes.

## Results

### Maternal Diabetes Induces the Loss of PDK1 in Mouse Oocytes

**FIGURE 1:**
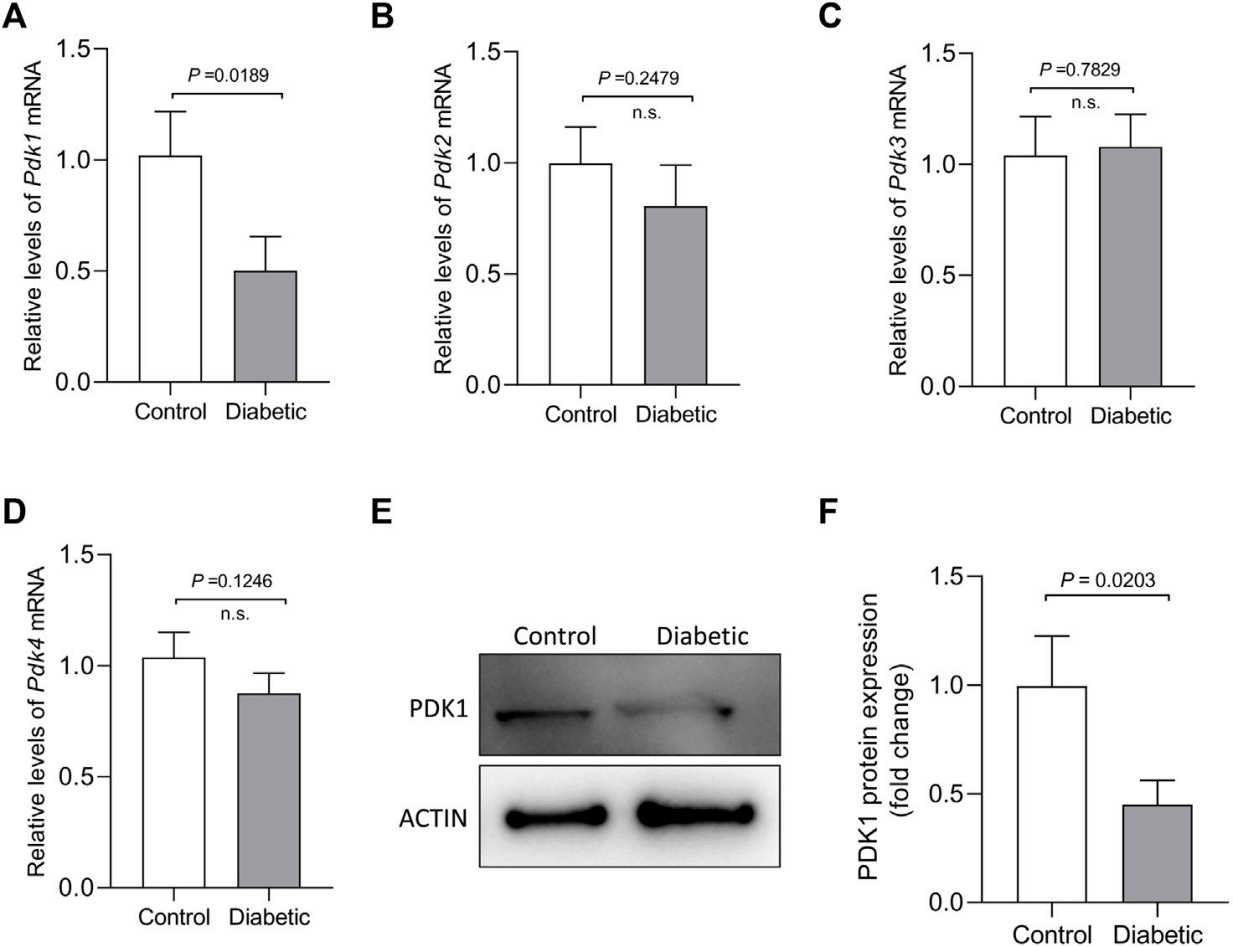
Reduced PDK1 expression in oocytes from diabetic mouse. **(A–D)** Quantitative RT-PCR analysis of *Pdks* mRNA levels in oocytes from control and diabetic mice. **(E–F)** Western blot analysis showed the decresed expression of PDK1 protein in oocytes from diabetic mice compared to controls. Actin served as an internal control. Band intensity was calculated using ImageJ software. Data are expressed as the mean percentage ± SD of three independent experiments.

PDKs, as key regulatory points in cellular metabolism, exhibit tissue- and cell-type-specific expression pattern. PDK1, one of the four PDKs, could inhibit phosphorylation on components of PDH complex that converts glycolysis to TCA cycle and lipogenesis in multiple tissues/cell types. Our previous work found that PDK1/2 are involved in spindle formation and chromosome movement in meiotic oocytes, partly through the phosphorylation of Ser232-PDHE1α (Hou et al., 2015). To explore the potential involvement of PDKs in the control of oocyte quality from diabetic mice, we first evaluated the expression of *Pdk* genes in fully-grown GV oocytes from control and diabetic mice. Remarkably, analysis of quantitative real-time PCR showed that the abundance of *Pdk1* mRNA was dramatically lowered in diabetic oocytes when compared to other three *Pdk* members (Figures 1A–D). Western blotting further confirmed that the expression of PDK1 protein was correspondingly reduced in diabetic oocytes (Figure 1E) relative to control oocytes. These observations strongly suggest that the PDK1 reduction may be associated with the poor oocyte quality derived from diabetic mice.

### Reduced Phosphorylation of Ser232-PDHE1α in Oocytes From Diabetic Mouse

**FIGURE 2:**
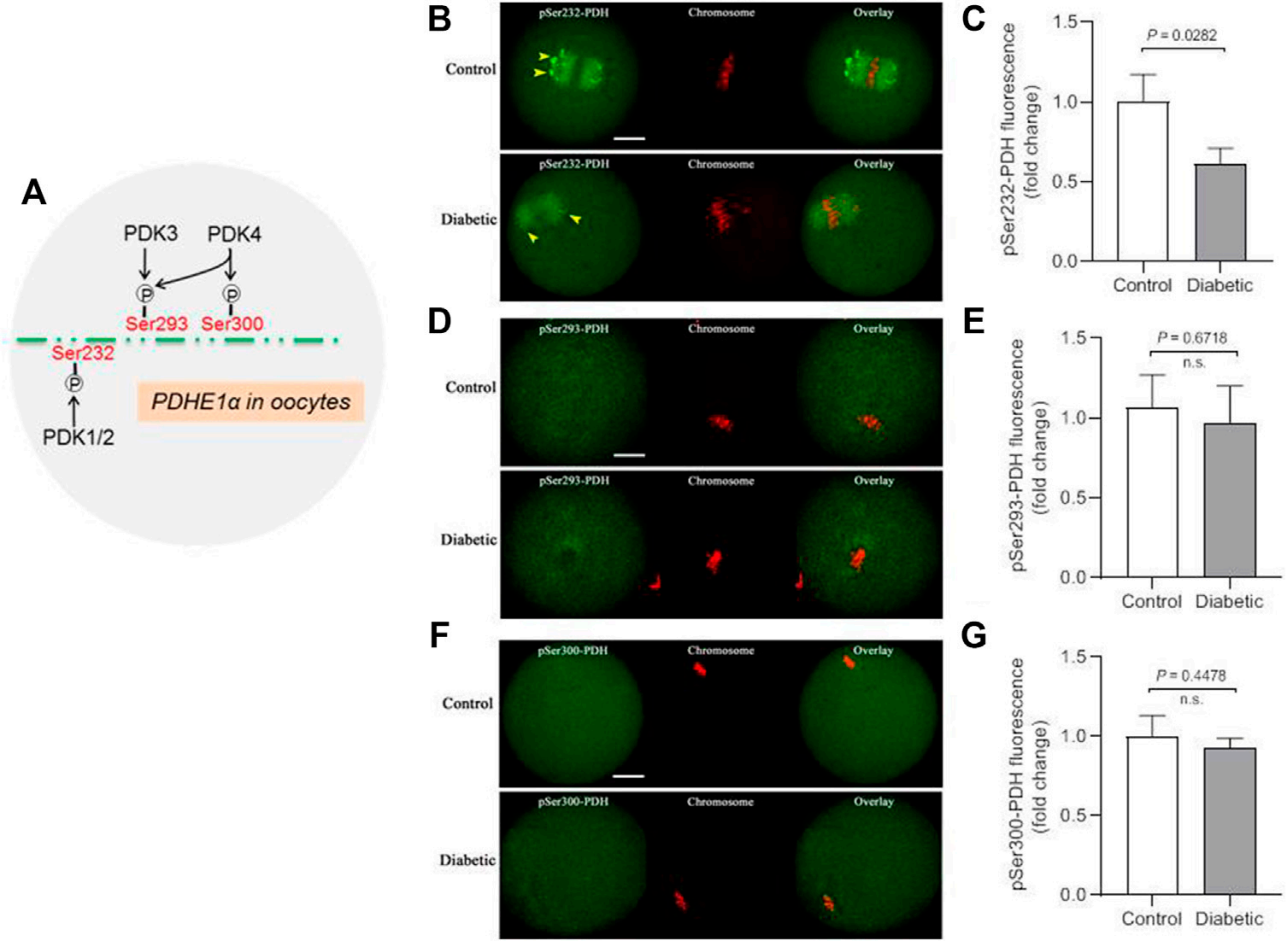
Lowered phosphorylation level of Ser232-PDHE1α in oocytes from diabetic mouse. **(A)** Schematic representation of the PDK paralog phosphorylation site specificity for oocyte PDHE1α. **(B)** Representative images of control and diabetic oocytes stained with antibodies against pSer232-PDHE1α (green) and co-stained with Propidium Iodide (PI, red) for chromosome. **(C)** Quantification of pSer232-PDHE1α fluorescence show in **(B)**. **(D)** Representative images of control and diabetic oocytes stained with antibodies against pSer293-PDHE1α (green) and co-stained with PI (red) for chromosome. **(E)** Quantification of pSer293-PDHE1α fluorescence show in **(D)**. **(F)** Representative images of control and diabetic mouse oocytes stained with antibodies against pSer300-PDHE1α (green) and co-stained with PI (red) for chromosome. **(G)** Quantification of pSer300-PDHE1α fluorescence show in **(F)**. Yellow arrowheads point to the pSer232-PDH signal in control and diabetic oocytes. Scale bar: 20 µm. At least 30 oocytes for each group were analyzed, and the experiments were repeated three times. Error bars indicate ± SD, a Student’s t test was used for statistical analysis. n.s., not significant.

PDKs have been demonstrated to be able to phosphorylate PDHE1α on three sites, Serine 232, Serine 293 and Serine 300 (Sugden and Holness 2003; Rardin et al., 2009). We previously revealed that each PDK isoform has specific action on these serine residues of PDHE1α in mouse oocytes. For example, PDK1/2 primarily regulates the phosphorylation state of Ser232-PDHE1α, whereas the phosphorylation state of Ser293-PDHE1α is mainly modulated by PDK3 (Figure 2A).

Thus, to examine the phosphorylation status of different serine residues on PDH complex, metaphase oocytes from control and diabetic mice were stained with a panel of antibodies against phosphorylated serine 232, 293 or 300 of PDHE1α subunit (hereafter indicates as pSer232-PDHE1α, pSer293-PDHE1α and pSer300-PDHE1α, respectively). As shown in Figure 2B, phosphorylated Ser232-PDHE1α exhibited a concentrated localization on the spindle region and its poles in meiotic oocytes (yellow arrowheads), consistent with our previous observation (Hou et al., 2015). It is worth noting that confocal scanning and quantitative analysis revealed a marked reduction of pSer232-PDHE1α in oocytes from diabetic mice. In contrast, maternal diabetes had little effects on the phosphorylation of Ser293-PDHE1α and Ser300-PDHE1α in oocytes (Figures 2D–F). Taking together, PDK1-mediated Ser232-PDHE1α phosphorylation is likely a critical pathway determining oocyte quality from diabetic mice.

### PDK1 Overexpression Ameliorates Meiotic Defects in Oocytes From Diabetic Mouse

**FIGURE 3:**
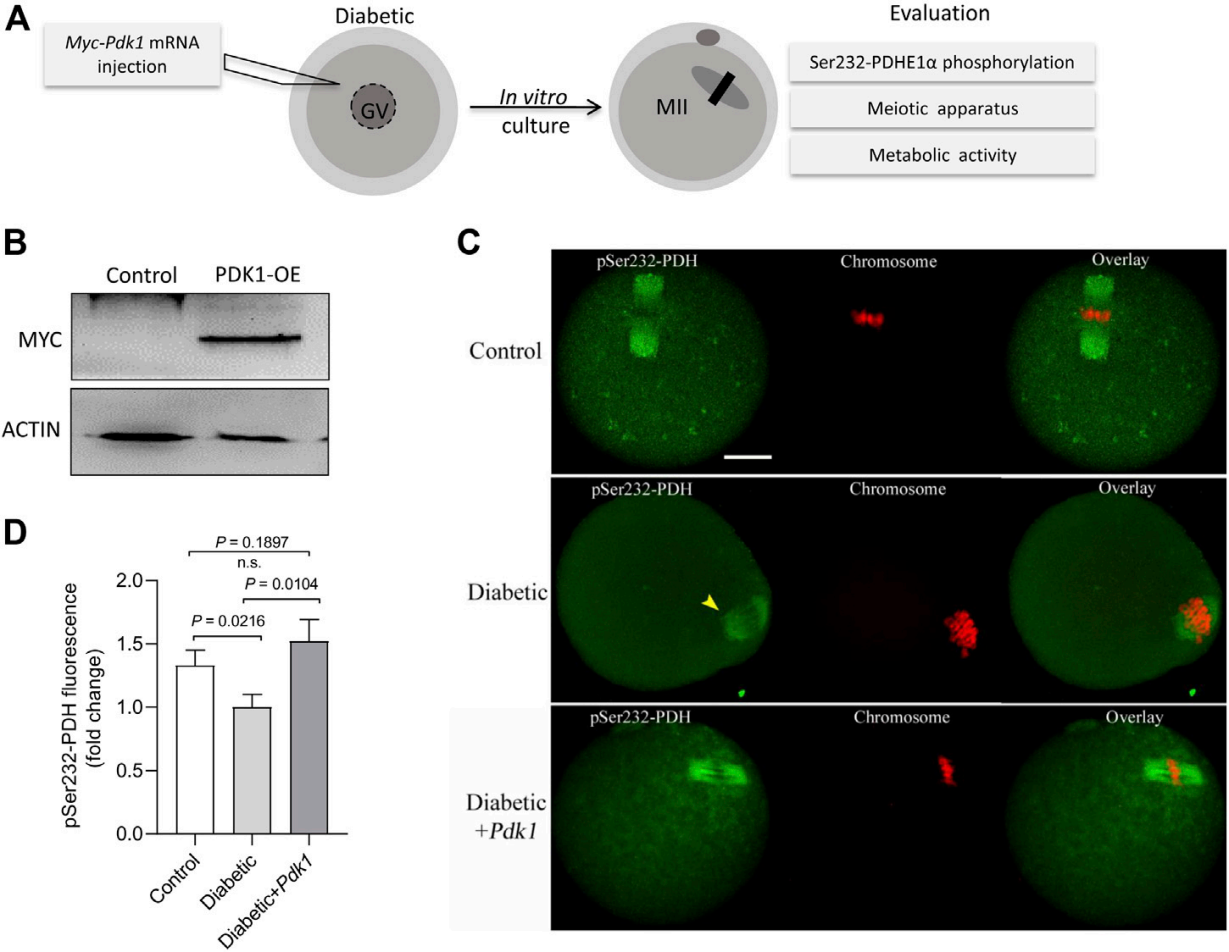
PDK1 overexpression increases the phosphorylation level of Ser232-PDHE1α in diabetic oocytes. **(A)** Diagram showing the experimental protocol to examine the effects of PDK1 overexpression on meiotic apparatus in diabetic oocytes. PBS or exogenous *Myc-Pdk1* mRNA was microinjected into GV oocytes. **(B)** Efficiency of PDK1 overexpression (PDK1-OE) after mRNA injection was confirmed by immunoblotting, probing with anti-MYC antibody. **(C)** Control, diabetic, and PDK1-overexpressing diabetic oocytes were stained with pSer232-PDHE1α antibody (green) and with PI for chromosomes (red). **(D)** Quantification of pSer232-PDHE1α fluorescence show in **(C)**. Scale bar: 20 µm. Error bars indicate ± SD, a Student’s *t* test was used for statistical analysis.

It has been widely reported that maternal diabetes induces a high frequency of spindle defects and chromosome misalignment in oocytes (Ratchford et al., 2008; Wang and Moley 2010; Wang et al., 2012). In PDK1/2-depleted oocytes, we consistently observed abnormal chromosome/spindle disorganization in metaphase oocytes (Hou et al., 2015). Therefore, we next asked whether elevating PDK1 expression is able to ameliorate the defective phenotypes of diabetic oocytes. To do this, overexpression experiments were carried out by injecting exogenous *Myc-Pdk1* mRNA into fully-grown GV oocytes that were arrested for 20 h with milrinone to allow synthesis of new PDK protein. After *in vitro*-culture in milrinone-free medium, matured oocytes (MII) were obtained for the following analysis (Figure 3A). Exogenous PDK1 protein was efficiently expressed as verified by immunoblotting (Figure 3B). Meanwhile, we also checked whether increased PDKs level in diabetic oocytes influences the phosphorylation status of Ser232-PDHE1α. As shown in Figures 3C,D, we found that ectopic expression of PDK1 significantly restored pSer232-PDHE1α staining in diabetic oocytes, providing additional support that PDK1 participates in the regulation of PDH phosphorylation in diabetic oocytes.

**FIGURE 4:**
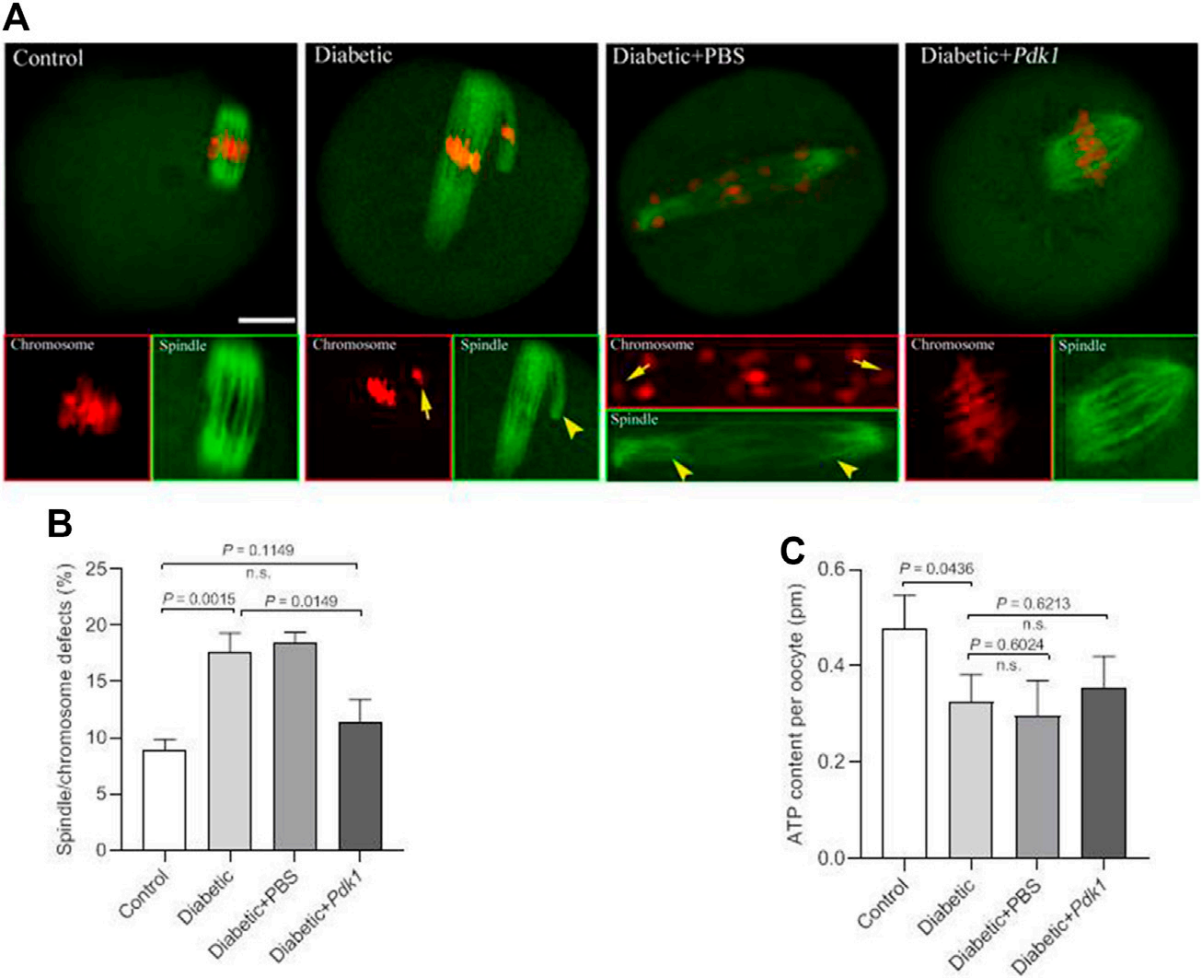
PDK1 overexpression alleviates meiotic defects in diabetic oocytes. **(A)** Control, diabetic, diabetic + PBS, and diabetic + PDK1 oocytes were labeled with α-tubulin antibody to show the spindle (green) and stained with PI to show chromosomes (red). Arrows indicate chromosome misalignment and arrowheads indicate spindle defect, respectively. Representative confocal images are shown. Scale bar: 20 µm. **(B)** Quantification of control, diabetic, diabetic + PBS, and diabetic + PDK1 oocytes with spindle/chromosome defects. Data are expressed as mean percentage ± SD from three independent experiments in which at least 120 oocytes were analyzed. n.s., not significant. **(C)** Measurement of total ATP content in control, diabetic, diabetic + PBS, and diabetic + PDK1 oocytes (*n* = 50 for each group). Data are expressed as mean percentage ± SD, student *t* test was used for statistical analysis. n.s., not significant.

In order to determine the effects of PDK1 overexpression on meiotic apparatus, MII oocytes were immunolabeled with anti-tubulin antibody to visualize the spindle and co-stained with propidium iodide to visualize chromosomes. Confocal microscopy revealed that most control metaphase oocytes presented a typical barrel-shaped spindle and well aligned chromosomes at the equatorial plate, while diabetic oocytes displayed a high frequency of diverse malformed spindles (*p* = 0.0015, Figure 4A). Quantitative analysis demonstrated that these meiotic defects in diabetic oocytes were significantly decreased when PDK1 expression was elevated (Figure 4B).

Pyruvate, the end-product of glycolysis, is ultimately destined for transport into mitochondria as a master fuel input undergirding TCA cycle carbon flux (Gray et al., 2014). Pyruvate metabolism by the PDH complex, with the product of pyruvate oxidation, acetyl-CoA and ATP serves as a main route for energy production in mammalian oocytes (Kim et al., 2006). To check whether PDK1 overexpression affects metabolic dysfunction in diabetic oocytes, total intracellular ATP level was measured by conducting microanalytical assay (Combelles and Albertini 2003). Maternal diabetes resulted in the diminished ATP content in mouse oocytes compared to controls (Figure 4C). Nonetheless, forced expression of PDK1 had little effects on ATP generation in oocytes (Figure 4C). Collectively, these findings provide the crucial evidence that loss of PDK1 protein is a major factor contributing to the assembly failure of meiotic apparatus in oocytes from diabetic mice.

### Phospho-Mimetic Mutant of Ser232-PDHE1α Partially Rescues Deficient Phenotypes of Oocytes From Diabetic Mice

**FIGURE 5:**
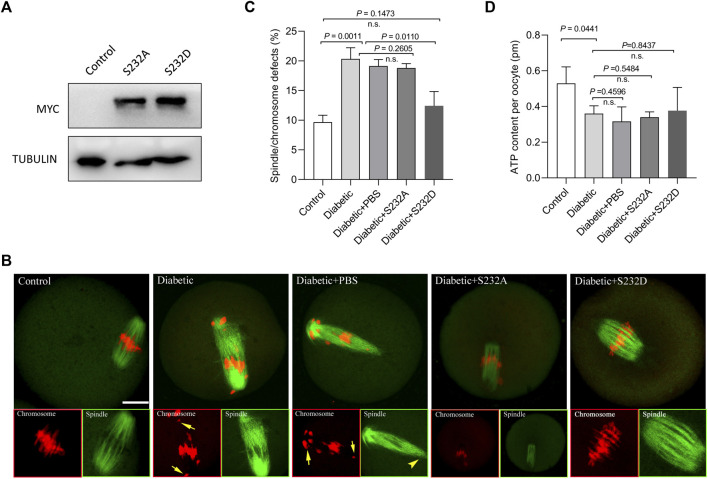
Phospho-mimetic mutant of Ser232-PDHE1α partially rescues deficient phenotypes of oocytes from diabetic mice. **(A)** Western blotting analysis showing that exogenous PDHE1α mutants were efficiently overexpressed, probing with anti-MYC antibody. **(B)** Control, diabetic, diabetic + PBS, diabetic + S232A-PDHE1α, and diabetic + S232D-PDHE1α oocytes were labeled with α-tubulin antibody to show the spindle (green) and stained with PI to show chromosomes (red). Arrows denote chromosome misalignment and arrowheads denote spindle defect, respectively. Scale bar: 20 µm. **(C)** Quantification of control, diabetic, diabetic + PBS, diabetic + S232A-PDHE1α, and diabetic + S232D-PDHE1α oocytes with spindle/chromosome defects. Data are expressed as mean percentage ± SD from three independent experiments in which at least 90 oocytes were analyzed. n.s., not significant. **(D)** Histogram showing the ATP level in control, diabetic, diabetic + PBS, diabetic + S232A-PDHE1α, and diabetic + S232D-PDHE1α (*n* = 50 for each group). n.s., not significant.

Moreover, we tested whether the phospho-mimetic mutant of Ser232-PDHE1α can rescue at least some of the phenotypic defects in diabetic oocytes with reduced expression of PDK1. For this purpose, we generated non-phosphorylatable mutants and phospho-mimetic mutants of Ser232-PDHE1α. Serine 232 was mutated to an alanine residue (S232A), to preclude phosphorylation, or to an aspartate residue (S232D), to mimic permanent phosphorylation (Vagnoni et al., 2011). S232A and S232D mutant mRNA was injected into fully-grown oocytes for spindle/chromosome analysis and ATP measurement. Immunoblotting verified that exogenous PDHE1α mutants were efficiently overexpressed in mouse oocytes (Figure 5A). In comparison to S232A-PDHE1α mutant, S232D-PDHE1α mutant significantly lowered the incidence of meiotic defects in diabetic oocytes (Figures 5B,C), close to the frequency of normal oocytes. In contrast, ectopic expression of S232D-PDHE1α mutant had no significant effects on the metabolism of diabetic oocytes, specifically the bulk ATP levels (Figure 5D). The results suggest that phospho-mimetic mutant S232D-PDHE1α is capable of protecting diabetic oocytes against meiotic defect, not metabolic dysfunction.

## Discussion

Maternal diabetes has been found to negatively influence embryonic development and pregnancy outcomes. A growing body of evidence has demonstrated that these effects are, at least in part, originated from the impaired oocyte quality (Wang and Moley 2010). In particular, spindle disorganization and chromosome misalignment were readily observed in oocytes from diabetic mice (Wang et al., 2009), consequently leading to the generation of aneuploid eggs. In addition, ovulated oocytes from diabetic mice displayed the altered mitochondrial ultrastructure, increased mitochondrial DNA copy number, reduced levels of ATP and TCA cycle metabolites, suggesting a mitochondrial metabolic dysfunction. However, to date, how maternal diabetes affects meiotic events in mammalian oocytes remains to be explored.

The pyruvate dehydrogenase complex (PDC) is a gatekeeper of glucose oxidation which links glycolysis to the TCA cycle by converting pyruvate to acetyl-CoA (Patel and Korotchkina 2006; Roche and Hiromasa 2007). Four PDKs isoenzymes are different with respect to their tissue distribution, kinetic parameters and regulation in mammals. Mounting evidence has suggested that PDKs are critical modulators of PDH activity in many cell types, through the phosphorylation of the PDH subunit at three sites on the E1 alpha subunit of PDH (PDHE1a): Ser232, Ser293, and Ser300 (Korotchkina and Patel 2001; Sugden and Holness 2003; Sugden and Holness 2006; Akhmedov et al., 2012). We previously identified that the divergent roles of PDKs during oocyte maturation (Hou et al., 2015). Intriguingly, in the present study, immunoblotting analysis revealed that the expression of PDK1 in diabetic mouse oocytes is less than that in the control (Figure 1). Importantly, PDK1 overexpression could partially rescue the spindle/chromosome defects in diabetic mouse oocytes. Furthermore, the phosphorylation level of pSer232-PDHE1α was markedly reduced in diabetic oocytes, while pSer293-PDHE1α and pSer300-PDHE1α signal remained unchanged (Figure 2). We discovered that PDK1/2 promotes the assembly of meiotic structure in mouse oocytes, likely by phosphorylating Ser232-PDHE1α, and which appears not to be through inactivation of PDH (Hou et al., 2015). In support of this concept, forced expression of exogenous PDK1 in diabetic oocytes restored the phosphorylation level of pSer232-PDHE1α, not the ATP content (Figure 3). Hence, our data identified PDK1 as a critical factor determining oocyte quality, and indicate that maternal diabetes environment disrupts PDK1-controlled meiotic structure in oocytes. Nonetheless, the mechanisms responsible for metabolic dysfunction, specifically insufficient ATP, in diabetic oocytes remain to be investigated in future research.

By performing *in vitro* kinase analysis, Korotchkina et al. found that all PDKs could phosphorylate Ser293 and Ser300, whereas PDK1 specifically phosphorylates Ser232 (Korotchkina and Patel 2001). Based on the observations from normal (Hou et al., 2015) and diabetic oocytes (Figure 3), we conclude that PDK3 controls phosphorylation of Ser293- PDHE1α in mouse oocyte, whereas PDK1/2 modulate the phosphorylation of Ser232-PDHE1α. By employing site-directed mutagenesis, we further revealed that ectopic expression of phospho-mimetic mutant of Ser232-PDHE1α alleviates the meiotic anomalies in diabetic oocytes (Figure 5). Together these data suggest that PDK1-controlled Ser232-PDHE1α phosphorylation participates in the protection of mouse oocytes, although this pathway is probably abnormally blunted in the diabetic model. Nevertheless, the present study is unable to exclude the possibility that other substrates or pathways might be simultaneously disturbed to impact meiosis in these oocytes. In addition, SIRT3-GSK3β deacetylation pathway has also been indicated to be involved in meiosis of diabetic oocytes (Xin et al., 2021). It is necessary to dissect the potential interaction between PDKs and SIRTs in mammalian germ cells in future research.

**FIGURE 6:**
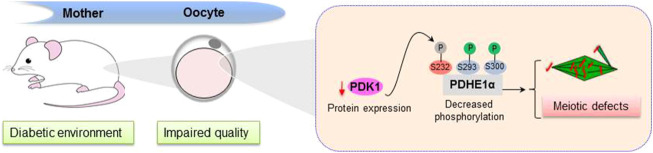
Diagram illustrating how PDK1 reduction affects oocyte quality from diabetic mice. Exposure of oocytes to maternal diabetes induces a significant reduction of PDK1, which results in a decreased in pSer232-PDHE1α, consequently leading to deficient spindle assembly and chromosome alignment in metaphase oocytes.

In summary, we propose a model where diabetes induces the PDK1 reduction in oocytes, which lowers the phosphorylation level of Serine 232 on PDHE1α and disturbs its enzymatic activity, consequently disrupting the assembly of meiotic apparatus during oocyte maturation (Figure 6). The present findings may have implications for preventing reproductive complications and birth defects.

## Materials and Methods

All chemicals and reagents were obtained from Sigma (St. Louis, MO, United States) unless otherwise stated.

### Animals

Female ICR mice were used in this study and maintained on a 12/12 h light/dark cycle at constant temperature (22°C) with specific pathogen-free conditions. All experiments were approved by the Animal Care and Use Committee of Nanjing Medical University. Diabetic mouse models were induced by a single intraperitoneal injection of streptozotocin (S0130; Sigma-Aldrich) at a dose of 190 mg/kg. Four days later, a tail-blood sample was measured for glucose concentrations. A diabetic model with glucose level higher than 300 mg/dl (16.7 mmol/L) was selected. Controls were injected with equal amount of sodium-citrate solution (S4641; Sigma-Aldrich).

### Antibodies

Rabbit polyclonal anti-PDK1 antibody was purchased from Abcam (Cambridge, MA, United States; Cat#: ab92959); Rabbit polyclonal anti-PDHE1α (pSerine232); Rabbit polyclonal anti-PDHE1α (pSerine293) and Rabbit polyclonal anti-PDHE1α (pSerine300) antibodies were purchased from EMD Chemicals (Beverly, MA, United States; Cat#: AP1063, AP1062 and AP1064); Myc-Tag Rabbit monoclonal antibody (cell signaling technology; Cat#:2278); Mouse monoclonal anti-β-actin and anti-α-tubulin-FITC antibodies were purchased from Sigma (St. Louis, MO, United States; Cat#: F2168 and A5441); FITC-conjugated goat anti-rabbit IgG antibody was purchased from Thermo Fisher Scientific (Rockford, IL, United States).

### Oocyte Collection and Culture

Control and diabetic mice were superovulated after intraperitoneal injections of 5 IU pregnant mare serum gonadotropin (PMSG). Cumulus-enclosed oocyte complexes were acquired by rupturing of antral ovarian follicles. Denuded oocytes were collected by removing cumulus cells with mouth-pipetting. Oocytes were *in vitro* cultured in M16 medium under mineral oil at 37°C in a 5% CO_2_ incubator.

### Quantitative Real-Time PCR

Quantitative RT–PCR was performed as described previously (Han et al., 2018). In brief, total RNA was extracted from 50 oocytes with an Arcturus PicoPure RNA Isolation kit (Applied Biosystems). The fold change in gene expression was calculated using the Comparative CT method (ΔΔCT). The house keeping gene, glyceraldehydes-3-phosphate dehydrogenase (GAPDH), was used as the internal control. Primer sequences are listed in Supplementary Table S1.

### Plasmid Construction and mRNA Synthesis

PDK1 plasmid construction was performed as we previously described (Zeng et al., 2018). Total RNA was obtained from 100 oocytes with Arcturus PicoPure RNA Isolation Kit (Applied Biosystems, CA, United States), and the cDNA was generated using QIA quick PCR Purification Kit (Qiagen, Germany). PCR products were digested with FseI and AscI (NEB Inc., MA, United States) after purification, and then cloned into the pCS2^+^ vector with Myc tags. The Myc-PDHE1α substitution mutants were produced by a QuickChange site-directed mutagenesis kit (Stratagene). For mRNA synthesis, the plasmids were linearized by NotI and capped RNAs were obtained with SP6 mMESSAGE mMACHINE (Ambion, CA, United States) based on the manufacturer’s instruction. Synthesized RNA was purified by RNeasy Micro Kit (Qiagen, Germany) and stored at −80°C. The primer sequences are listed in Supplementary Table S2.

### Overexpression Experiment

Microinjection was performed using an inverted microscope (Eclipse Ti-S, Nikon) equipped with a micromanipulator (Narishige) as we described previously (Zeng et al., 2018). For overexpression analysis, 10 pL mRNA solution (10 ng/µL) was injected into cytoplasm of fully-grown oocytes. The same amount of RNase-free PBS was injected in control oocytes.

### Immunofluorescence

Following fixation with 4% paraformaldehyde for 30 min, oocytes were permeabilized with 0.5% Triton X-100 for 20 min. Following incubation in 1% BSA blocking buffer for 1 h at room temperature, oocytes were incubated with FITC-conjugated anti-tubulin antibody overnight at 4°C. Chromosomes of oocytes were evaluated by co-staining with propidium iodide (red) for 10 min. Samples were examined using a laser scanning confocal microscope (LSM 700; Zeiss, Oberkochen, Germany). The intensity of fluorescence was measured and quantified using ImageJ software (NIH), as we described previously (Han et al., 2018).

### Western Blot Analysis

Samples (100–150 oocytes) were lysed in Laemmli sample buffer with protease inhibitor, then denatured at 100°C for 5 min. Protein extracts were separated by SDS-PAGE gel and electrically transferred to polyvinylidene fluoride (PVDF) membranes. Followed by blocking with 5% skimmed milk, membranes were incubated with anti-PDK1 antibody (1:1000) overnight at 4°C. After multiple washes, protein samples were incubated with anti-rabbit horseradish peroxidase conjugated antibody for 1 h at room temperature. The protein bands were visualized using an ECL Plus Western Blotting Detection System (GE Healthcare, Piscataway, NJ, United States).

### Determination of ATP Content

Pools of 15 oocytes were used for measuring ATP content with bioluminescent somatic cell assay kit (Sigma, MO, United States), according to the published protocol (Combelles and Albertini 2003). A 5-point standard curve (0, 0.1, 0.5, 1.0, 10, and 50 pmol) was generated in each assay. The formula derived from the linear regression of standard curve was employed to calculate the ATP levels in each oocyte.

## Statistical Analysis

All experiments were repeated at least three times. Data are presented as mean ± SD, unless otherwise indicated. Statistical comparisons were made with Student’s *t* test and one-way ANOVA test when appropriate Prism 5.0 (GraphPad, San Diego, CA, United States). Changes were considered statistically significant when *p* < 0.05.

## Data Availability

The original contributions presented in the study are included in the article/Supplementary Material, further inquiries can be directed to the corresponding author.
