# Supplementary material for: Loss of PDK1 Induces Meiotic Defects in Oocytes From Diabetic Mice
Source: Front Cell Dev Biol. 2021 Dec 20;9:793389. doi: 10.3389/fcell.2021.793389 (PMC8720995; doi:10.3389/fcell.2021.793389)
Supplement: Supplementary file 1 [file Table1.DOCX]

**Table S1 Primer sequences of genes for qRT-PCR**

***Gene Primer sequence***

GAPDH Forward Primer: 5’ –CTTTGTCAAGCTCATTTCCTGG – 3’

Reverse Primer: 5’ –TCTTGCTCAGTGTCCTTGC – 3’

PDK1 Forward Primer: 5’ –GACTGTGAAGATGAGTGACCG – 3’

Reverse Primer: 5’ –CAATCCGTAACCAAACCCAG – 3’

PDK2 Forward Primer: 5’ –AAGAGATCAACCTGCTTCCTG – 3’

Reverse Primer: 5’ –GCATCTGTGAACTGGCTTAGAG – 3’

PDK3 Forward Primer: 5’ –CGCCATTACAAGACCACTCC– 3’

Reverse Primer: 5’ –CAGAGACTTCAGAGACAGCAC– 3’

PDK4 Forward Primer: 5’ –AGTGACTCAAAGACGGGAAAC– 3’

Reverse Primer: 5’ –GTGTGAGGTTTAATTCTGGCG – 3’
