# Supplementary material for: Loss of PDK1 Induces Meiotic Defects in Oocytes From Diabetic Mice
Source: Front Cell Dev Biol. 2021 Dec 20;9:793389. doi: 10.3389/fcell.2021.793389 (PMC8720995; doi:10.3389/fcell.2021.793389)
Supplement: Supplementary file 2 [file Table2.DOCX]

**Table S2 Primer sequences of genes for site-directed mutagenesis of PDHE1a**

***Gene Primer sequence***

PDH-Ser232A Forward Primer: 5’ –CTATGGCATGGGGACGGCTGTTGAGAGAGCAGC – 3’

Reverse Primer: 5’ –GCTGCTCTCTCAACAGCCGTCCCCATGCCATAG – 3’

PDH-Ser232D Forward Primer: 5’ –CTATGGCATGGGGACGGATGTTGAGAGAGCAGCAG – 3’

Reverse Primer: 5’ –CTGCTGCTCTCTCAACATCCGTCCCCATGCCATAG– 3’
